# Probiotics: Can it modulate fracture healing?

**DOI:** 10.1371/journal.pone.0290738

**Published:** 2023-08-31

**Authors:** Yufa Wang, Aouod Agenor, Allison Clement, Adam Hopfgartner, Cari Whyne, Diane Nam

**Affiliations:** 1 Sunnybrook Research Institute, Toronto, ON, Canada; 2 Department of Surgery, University of Toronto, Toronto, Canada; 3 Institute for Biomedical Engineering, University of Toronto, Toronto, Canada; University of Vermont College of Medicine, UNITED STATES

## Abstract

**Objective:**

Fractures remain a huge burden and their management adversely affects individuals’ function and productivity during the lengthy healing period. Gut microbiota exerts a systemic influence on diverse aspects of host physiology, including bone. The primary objective of this study was to evaluate if oral probiotic treatment before or after a fracture in a mouse model could increase cytokines and biomarkers essential for bone healing with subsequent improvement in the biomechanical properties of the healed callus.

**Methods:**

Femoral osteotomy and intramedullary pinning were performed on C57BL/6 mice. Group 1 received either control PBS or probiotic via oral gavage for 5 weeks before fracture (pre-fracture). Group 2 received equivalent treatments for 4 weeks only after fracture (post-fracture). Fracture calluses were harvested on day 3 and 7 for RT-qPCR to quantify osteogenic-related inflammatory cytokines and bone biomarkers. Fractured femurs were evaluated day 28 post-osteotomy via microstructural analysis (μCT) and biomechanical testing (torsion).

**Results:**

Mice treated with probiotics pre-fracture (group 1) showed significantly increased gene expression on day 3 of cytokines TGF-β, IL-6 and IL-17F and a corresponding increase in gene expression on day 7 for Col1 and Runx2. Significant improvement was also seen in bone volume fraction, bone mineral density, tissue mineral density, maximum yield torque, stiffness and strain energy.

Mice treated with probiotics post-fracture (group 2), demonstrated no changes in cytokine or bone marker gene expression with no significant changes on microstructural analysis. However, significant increases were seen in twist angle at failure and strain energy, with a corresponding reduction in torsional stiffness.

**Conclusion:**

Our results suggest that oral probiotic administration, before or after a fracture, may sufficiently alter the gut flora microenvironment leading to improved bone healing biomechanical properties. The use of probiotics may provide a cost-effective and low-risk adjunctive therapy to improve fracture healing.

## Introduction

Bone healing at the cellular level is a complex biochemical process requiring the regulation of multiple cytokines and structural proteins [1]. We previously established that IL-17F is an important regulator of fracture healing and a key pro-inflammatory immune mediator in osteoblast maturation [2–4]. In the early inflammatory phase of the immune response to fracture injury, we determined that IL-17F is secreted by T-helper cell 17 and expressed in the fracture hematoma [3]. IL-17F was found to bind to its IL-17F receptor on osteoblasts upregulating Runx2, a key transcription factor associated with osteoblast differentiation [5] and directly induced by IL-17F. Other proteins shown to be intimately related to robust bone healing include TGF- β [6] (osteoblast differentiation or proliferation inducer) and type 1 collagen (primary collagen found in bone). Controlled regulation of these cytokine pathways could potentially lead to an optimal milieu for bone healing with expedited callus formation and tougher bone [1].

Gut microbiota exerts a systemic influence on diverse aspects of host physiology. This rapidly growing area of research demonstrates the importance of microbiomes for intestinal barrier function, energy metabolism, immune and inflammatory responses as well as for disease prevention and treatment [7,8]. Interest in the effect of gut microbiota on cytokine pathways, including those involved in bone remodeling, and biomechanical properties has been evolving. Manipulation of microbiota by probiotic supplementation has been shown to alter bone remodeling, development, growth, and mechanical strength [9,10]. Multiple mechanisms have been postulated such as modulating membrane integrity, increasing mucins as well as affecting the inflammatory cells such as modulating T-regulators, B cells and CD4+ cells [7]. However, studies assessing its potential efficacy to improve fracture healing in the acute setting are lacking. It is unknown whether probiotic utilization pre-fracture, or administration post-fracture, will improve bone healing.

The objective of this study was to demonstrate the impact of probiotic ingestion on long bone fracture healing when administered 1) pre-fracture or, 2) post-fracture to determine the impact of a commercially available oral probiotic to regulate gene expression of pro-inflammatory cytokines and mature bone biomarkers critical to bone healing and affect the fracture callus properties under biomechanical testing in a healthy mouse fracture model.

## Material and methods

### Animal model

All animal experiments were approved by our institutional Animal Care Committee. A total of 95 C57BL/6 male mice were used. At 11 weeks of age, the right femur was pre‐stabilized with an intramedullary pin and a fracture was created via mid-shaft open osteotomy performed through a small lateral approach. The animals were allowed free, unrestricted weight-bearing in their cages with full access to food and water immediately following recovery from anesthesia. Animals were sacrificed and femurs were harvested at three time-points, days 3, 7 and 28 after fracture.

### Treatment and harvest

**Fig 1:**
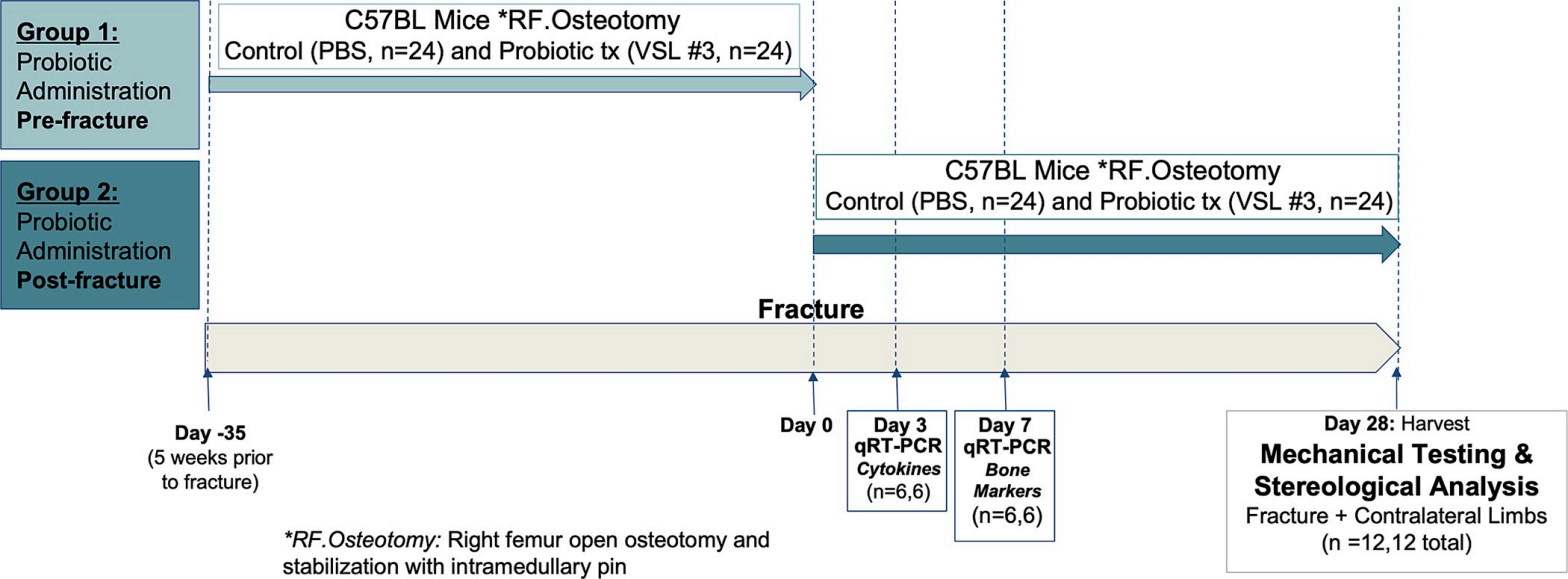
Proposed experimental fracture model to determine the effects of probiotic treatment pre-fracture (Group 1) vs. probiotic treatment post-fracture (Group 2).

In group 1, probiotic was administered for 5 weeks before fracture (timing and duration based on pilot work) and in group 2, probiotic treatment was administered for 4 weeks after the fracture (minimum standard time to heal mice femur fractures). In each group, mice were assigned to one of two treatments for daily oral gavages: control volumetric equivalent of Phosphate Buffered Solution (PBS) (N = 24) or 1x10^9^ CFU of probiotic VSL#3 (N = 24). VSL#3 is a proprietary blend by Actial Farmaceutica (Rome, Italy) combining several bacterial strains (*Lactobacillus acidophilus*, *Lactobacillus plantarum*, *Lactobacillus casei*, *Lactobacillus delbrueckii* subspecies *bulgaricus*, *Bifidobacterium breve*, *Bifidobacterium longum*, and *Bifidobacterium infantis* and *Streptococcus salivarius* subspecies *thermophilus*). Its efficacy has been shown in many gastroenterology clinical studies [11]. Probiotic microbes are not metabolized but merely present in the gut, therefore a quantity of 1x10^9^ CFU was chosen as it is equivalent to a weight-based calculation of typical human consumption of probiotics (~900x10^9^ to 2000x10^9^ CFU) in previous studies involving VSL#3 [12,13]. Probiotics were administered once daily Monday to Friday until harvest (no treatment on weekends). Previous studies also utilized intermittent breaks in probiotic treatment to rest the gut flora [14]. Six animals per group were sacrificed on days 3 and 7 post fracture for biomolecular analysis. Twelve animals per group were sacrificed 28 days post fracture to evaluate bone healing through microstructural assessment and biomechanical testing. The callus from both fractured and the contralateral intact femurs were harvested, frozen in liquid nitrogen and stored at −80°C. The femurs for microstructural / biomechanical analyses were stored at −20°C. Protocol outline is shown in Fig 1.

#### Fracture callus biomolecular analysis

RT-qPCR analysis was performed to quantify mRNA expression of osteogenic-related inflammatory markers in the fracture hematoma / callus at day 3 and mature bone marker expression at day 7, representing previously reported peak levels in fracture healing [1,4–6]. These markers generally return to normal levels 2 weeks post-fracture.

A tissue homogenizer was used for RNA extraction and processing due to the calcified matrix and relatively low cellular yield of bone. The liquid nitrogen frozen callus was pulverized by BioPulverizer (MidSci, Fenton, MO) and total RNA extracted using TRIZOL (Invitrogen, Waltham, MA). Quantitative RT-qPCR performed with a StepOnePlus system (Applied Biosystems, Waltham, MA) using SYBR Green (Bio-Rad, Hercules, CA). Pro-inflammatory cytokine primers Transforming Growth Factor Beta (TGF-β), interleukin 6 (IL-6) and interleukin 17F (IL-17F), and bone marker primers, collagen 1 (Col1), collagen 2 (Col2), and Runt-related transcription factor 2 (Runx2) were assessed. GAPDH was used as a baseline for comparative analysis. Primer sequences can be found in Table 1. Of note, we previously established in this fracture model that inflammatory cytokine and bone marker were only upregulated in response to injury at early timepoints, with analyses of additional timepoints at day 14, 21, and 28 post-fracture demonstrating no detectable differences3. As such inflammatory cytokine and bone marker analyses in this study were confined to early days 3 and 7 timepoints. The rationale for the choice of cytokines TGF-β, IL-6, IL-17F and IL-10 is based on our previous work [3] that demonstrated these specific cytokines as being significantly up-regulated in the fracture callus of C57BL/6 mice implicating their importance in the regulation of fracture healing.

**Table 1:** List of primers.

No.	Gene		Sequence(5’ to 3”)
**1**	GAPDH	Forward Reverse	AACTTTGGCATTGTGGAAGG ACACATTGGGGGTAGGAACA
**2**	TGFβ	Forward Reverse	TTGCTTCAGCTCCACAGAGA TGGTTGTAGAGGGCAAGGAC
**3**	IL-6	Forward Reverse	AGTTGCCTTCTTGGGACTGA TCCACGATTTCCCAGAGAAC
**4**	IL-17F	Forward Reverse	GTGTTCCCAATGCCTCACTT GTGCTTCTTCCTTGCCAGTC
**5**	Col1	Forward Reverse	GAGCGGAGAGTACTGGATCG GCTTCTTTTCCTTGGGGTTC
**6**	Col2	Forward Reverse	GCCAAGACCTGAAACTCTGC GCCATAGCTGAAGTGGAAGC
**7**	Runx2	Forward Reverse	CCCAGCCACCTTTACCTACA TATGGAGTGCTGCTGGTCTG

#### Imaging and microstructural analysis

Harvested femurs were thawed at 4°C for 24 h before imaging. Scans were reconstructed using NRecon software and processed in AmiraDEV 5.3 (Visage Imaging, Carlsbad, CA). A region of interest (ROI) was defined for each femur starting below the lesser trochanter and ending at the epicondyles. An intensity‐based minimum threshold value of 365 mgHA/cm3 was applied to remove unmineralized callus, leaving an ROI containing mineralized callus and original cortical bone. Standard microstructural measures of bone volume (BV), total volume (TV), bone volume fraction (BV/TV), bone mineral density (BMD), and tissue mineral density (TMD) were computed using CT Analyser software (SkyScan, Kontich, Belgium) [15].

#### Biomechanical testing

Harvested femurs were mechanically tested under torsional loading on a Bionix 858 materials testing system (MTS Systems, Eden Prairie, MN). The regions below the lesser trochanter and above the epicondyles were potted in bone cement, leaving a 10mm working length. Torque was measured using a 1.4 Nm Futek reaction torque transducer during the application of angular displacement (1°/second) until failure (identified by a sudden drop in torque) or to a maximum displacement of 30° [16,17]. Yield point was identified in the resulting load deformation curve, and the maximum yield torque and twist angle at failure determined. Torsional stiffness was calculated as the linear slope of the elastic region and the strain energy as the area under the load-deformation curve.

#### Statistical analysis

Data are expressed as mean ± standard deviation. The probiotic treated groups and their respective control groups were compared using unpaired Student’s t‐tests. P≤0.05 was considered statistically significant.

## Results

### Probiotic treatment pre-fracture increases key bone biomarker gene expression and improves callus mineral density and biomechanical properties in healing mice femurs

**Fig 2:**
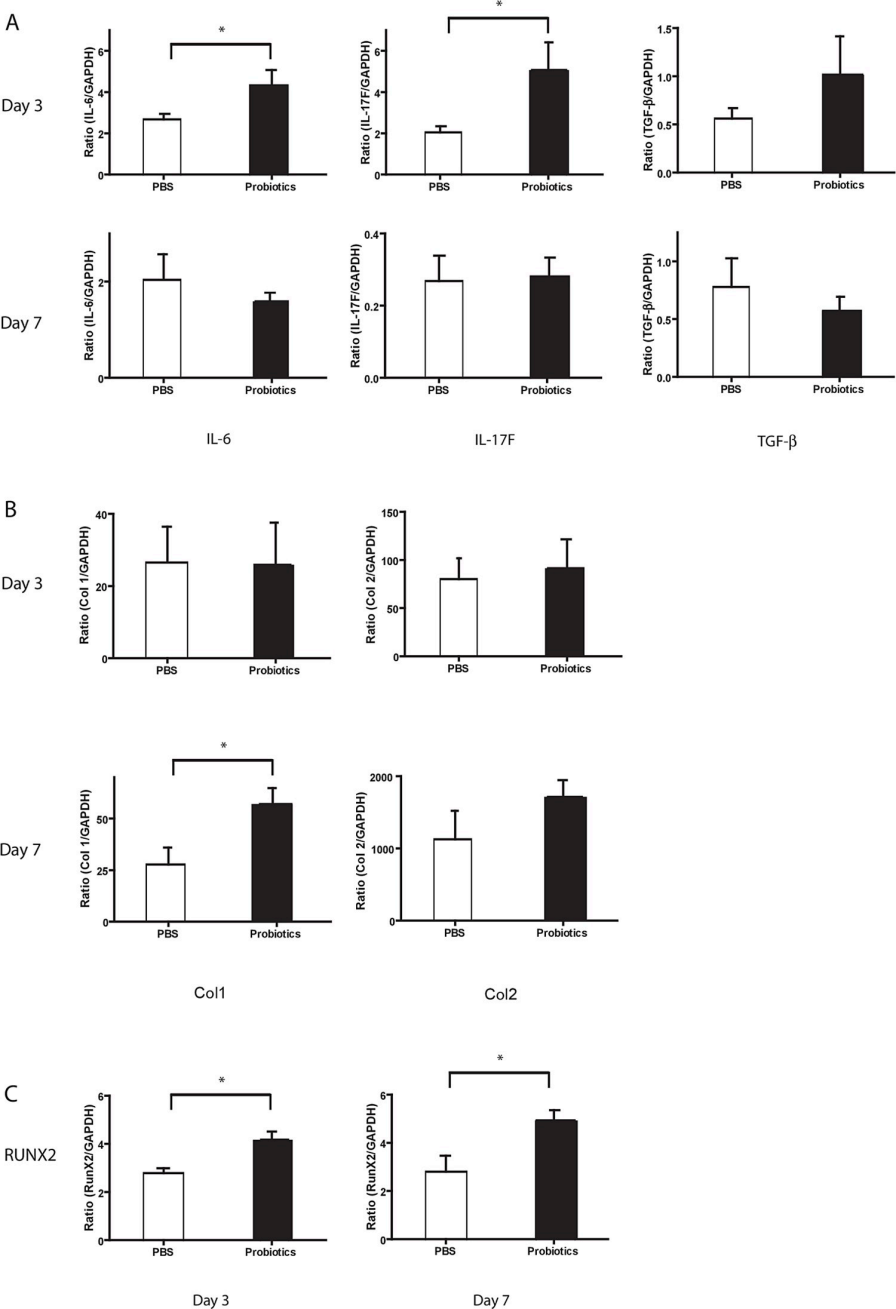
Group 1 probiotic treatment pre-fracture. A: Cytokine gene expression at day 3 after fracture using quantitative RT-PCR demonstrated increase in pro-inflammatory cytokines IL-6 and IL-17F (*P<0.05) with no difference in the expression of the anti-inflammatory cytokine TGF-β. No differences were seen at day 7. B: Bone marker gene expression (Col1, Col2 –Type 1, Type 2 Collagen) expression was only increased at day 7 after fracture for Col1 with no differences seen on day 3. Results showed VSL#3 promoted expression of Col1 at day 7. *P<0.05. C: Gene expression of transcription factor RUNX2 was elevated at day 3 and day 7 after fracture. *P<0.05. Means are presented ±SEMs.

Group 1 mice treated 5 weeks with probiotics pre-fracture showed a significant increase in key pro-inflammatory cytokine and bone marker gene expression in the fracture callus on day 3 compared to untreated controls. IL-6 (probiotic 4.33±0.74 vs. control 2.67±0.27) and IL-17F (critically involved in osteoblastogensis by increasing mature osteoblasts, 5.07±1.34 vs. 2.04±0.30) were significantly higher but no significant difference was noted for TGF-β (0.64±0.11 vs 0.56±0.15, P = 0.46) (Fig 2A). These results align with a significant increase of mature bone biomarker gene expression of Col1 on day 7 (57.01±7.74 vs 27.75±8.13). No difference was noted for Col2 (primarily cartilage) (1716±230 vs 1128±394, P = 0.24) (Fig 2B). Runx2 on days 3 and 7 was significantly upregulated (4.17±0.34 vs 2.78±0.21 and 4.94±0.42 vs 2.80±0.66) (Fig 2C). These results suggest that regulation in the gene expression of inflammatory mediators and bone markers involved in the early phase of the fracture healing process may be related to changes in the gut microbiota environment following probiotic administration.

**Fig 3:**
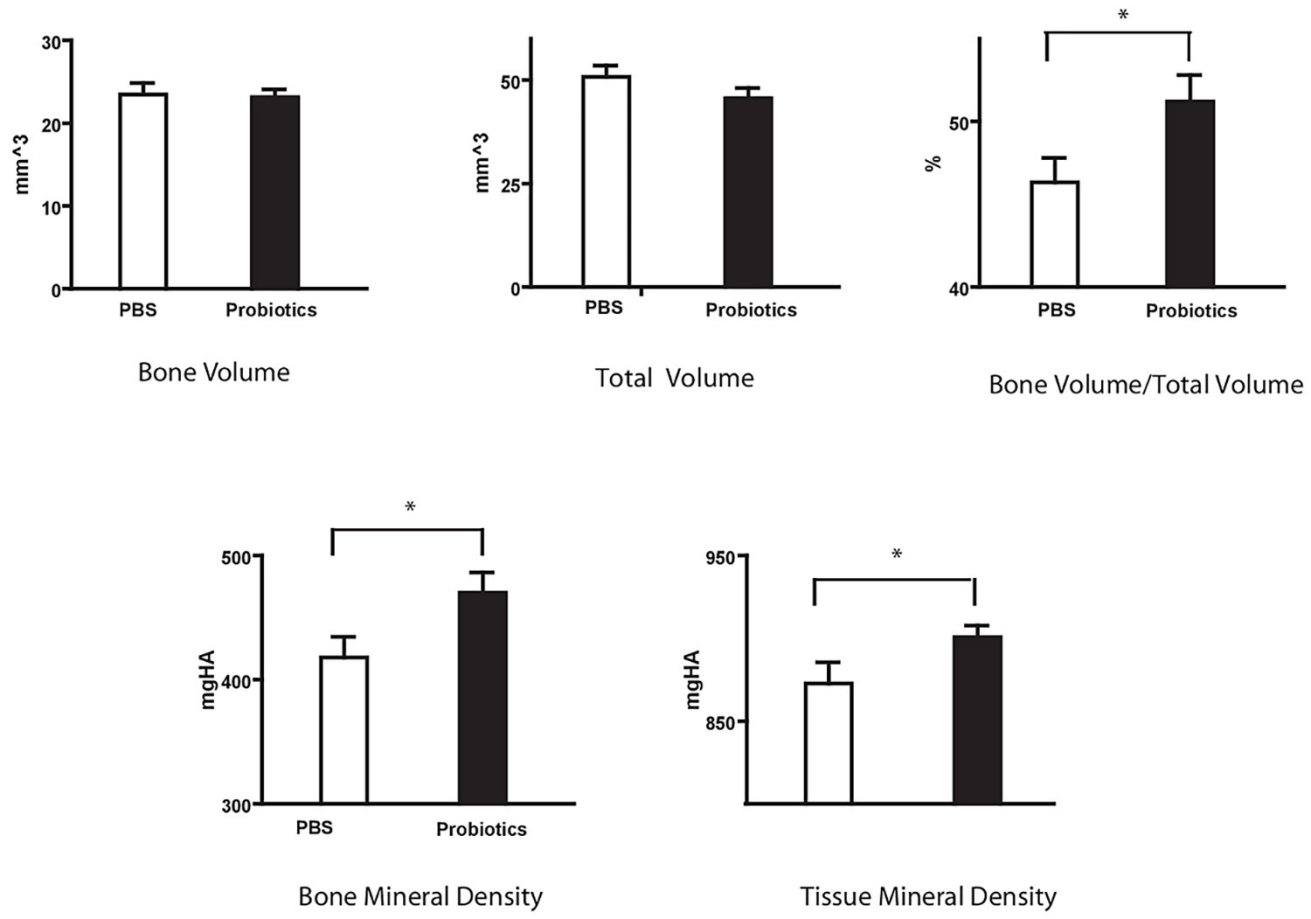
Group 1 stereologic analysis of fractured limb callus at day 28 after fracture—μCT analysis confirmed significantly higher bone mineral density and tissue mineral density in the probiotic treatment pre-fracture group compared to control with increase in bone volume fraction. *P<0.05.

Group 1 mice microstructural analysis on day 28 post fracture also demonstrated significant increases in the probiotic group with respect to bone volume/total volume (BV/TV) (probiotic 51.21±1.60 vs control 46.33±1.47), bone mineral density (BMD) (470.08±16.30 vs 417.86±16.74 mgHA/mm^3^) and tissue mineral density (TMD) (900.71±6.94 vs 872.68±12.31 mgHA/mm^3^). Individual fracture callus size represented by BV (23.13±0.94 vs 23.47±1.38 mm^3^, P = 0.42) and TV (45.60±2.47 vs 50.77±2.76 mm^3^, P = 0.09) were not statistically different (Fig 3).

**Fig 4:**
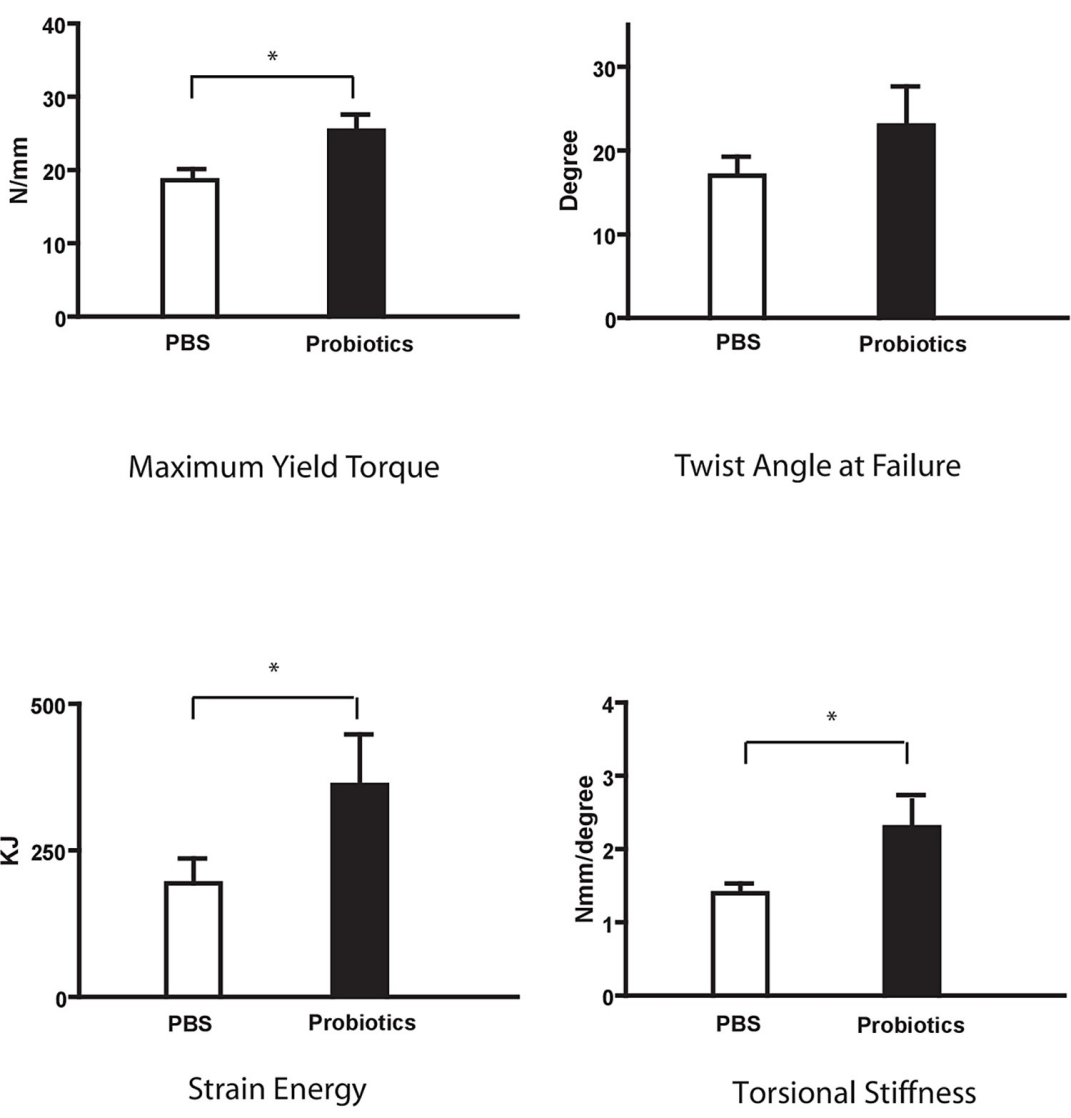
Group 1 biomechanical testing of fractured limb callus at day 28 after fracture–Mechanical assessment of samples showed significantly higher maximum yield torque, strain energy and torsional stiffness in the probiotic treatment pre-fracture group *P<0.05.

Biomechanical testing showed that the probiotic group bone had significantly higher maximum yield torque (25.44±2.18 vs 18.62±2.18 N-mm), torsional stiffness (2.3±0.36 vs1.4±0.13 N-mm/°) and strain energy (361.8±86.2 vs 194.2±42.5 KJ) (Fig 4). There was no significant difference in twist angle at failure (22.98±4.66° vs 16.98±2.66°, P = 0.13).

#### Probiotic treatment post-fracture only improves the biomechanical properties in healing mice femurs

**Fig 5:**
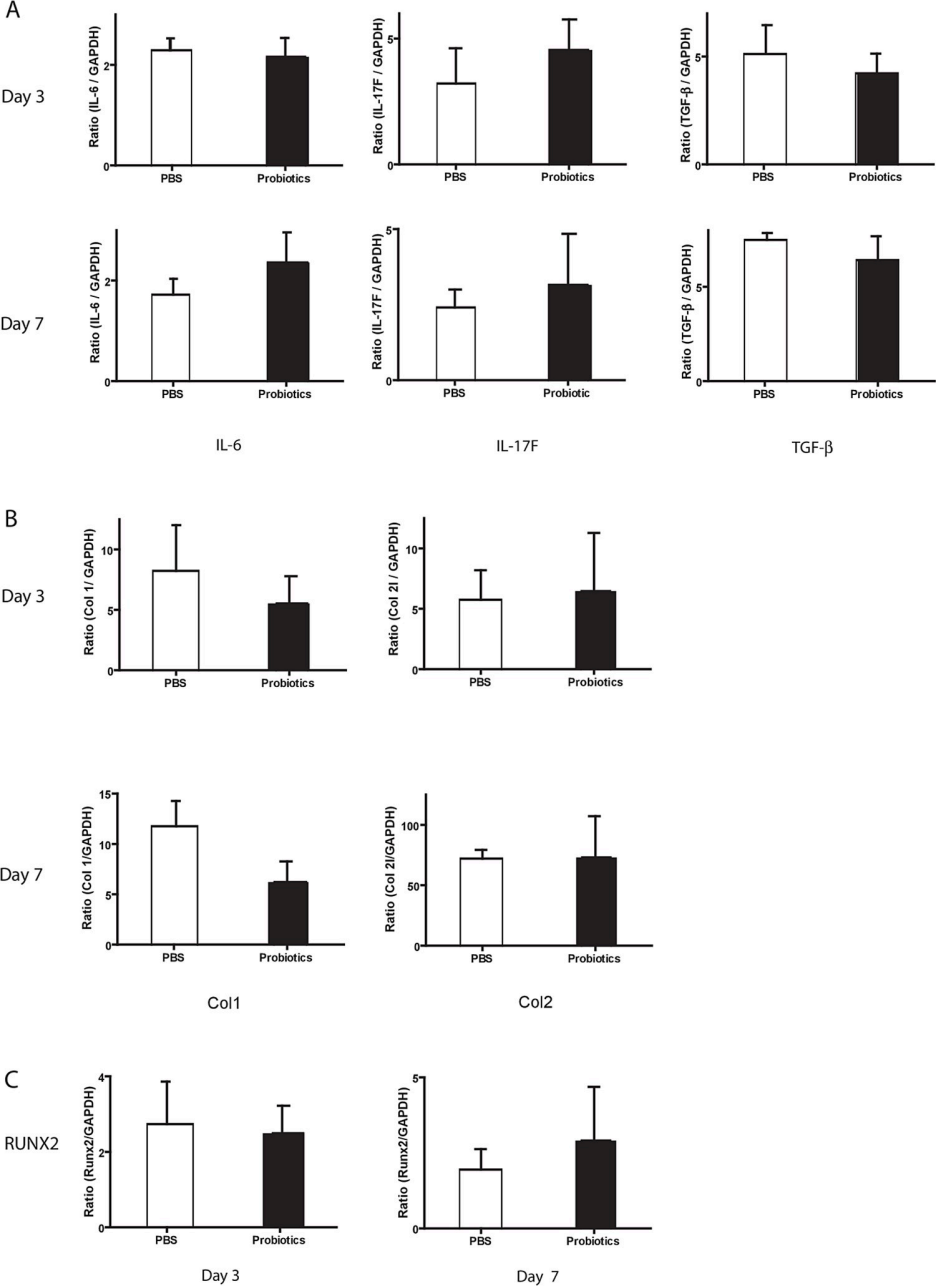
Group 2 probiotic treatment post-fracture–No significant differences were detected in the gene expression of pro-inflammatory cytokines or mature bone markers in the fracture callus on day 3 and 7 compared to untreated controls P>0.05. Means are presented ±SEMs.

Initiating probiotic treatment prior to unexpected traumatic fracture in the clinical setting is not possible. In considering whether administering oral probiotics at the time of fracture/injury would still be able to provide a beneficial bone healing response in the absence of a primed gut microbiome reaching a steady state, the group 2 mice were treated for 4 weeks with probiotics post-fracture. Our results for probiotic treatment post-fracture showed no significant difference in pro-inflammatory cytokine or bone marker gene expression in the fracture callus on day 3 and 7 compared to untreated controls (Fig 5).

**Fig 6:**
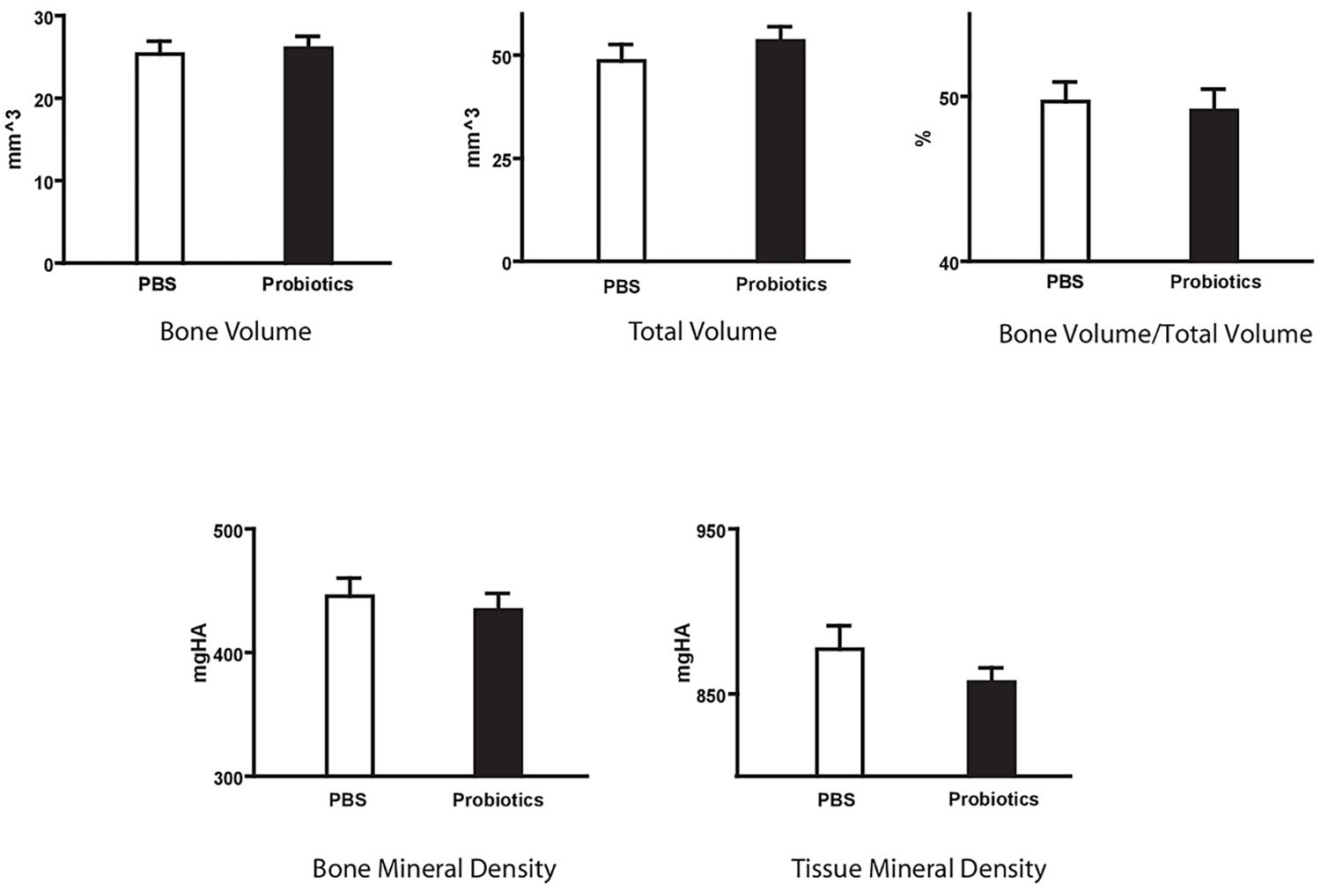
Group 2 stereologic analysis of fractured limb callus at day 28 after fracture—μCT analysis confirmed no differences in bone volume, total volume, bone mineral density and tissue mineral density in the probiotic treatment post-fracture group compared to control. P>0.05.

Group 2 microstructural analysis at day 28 post fracture also demonstrated no significant differences between probiotic treated and non-treated controls with respect to BV (26.64±1.44 vs 25.35±1.56 mm^3^, P = 0.28), BV/TV (49.14±1.30 vs 49.69±1.19%, P = 0.30) or BMD (434.44±13.55 vs 445.48±14.78 mgHA/mm^3^, P = 0.08) (Fig 6).

**Fig 7:**
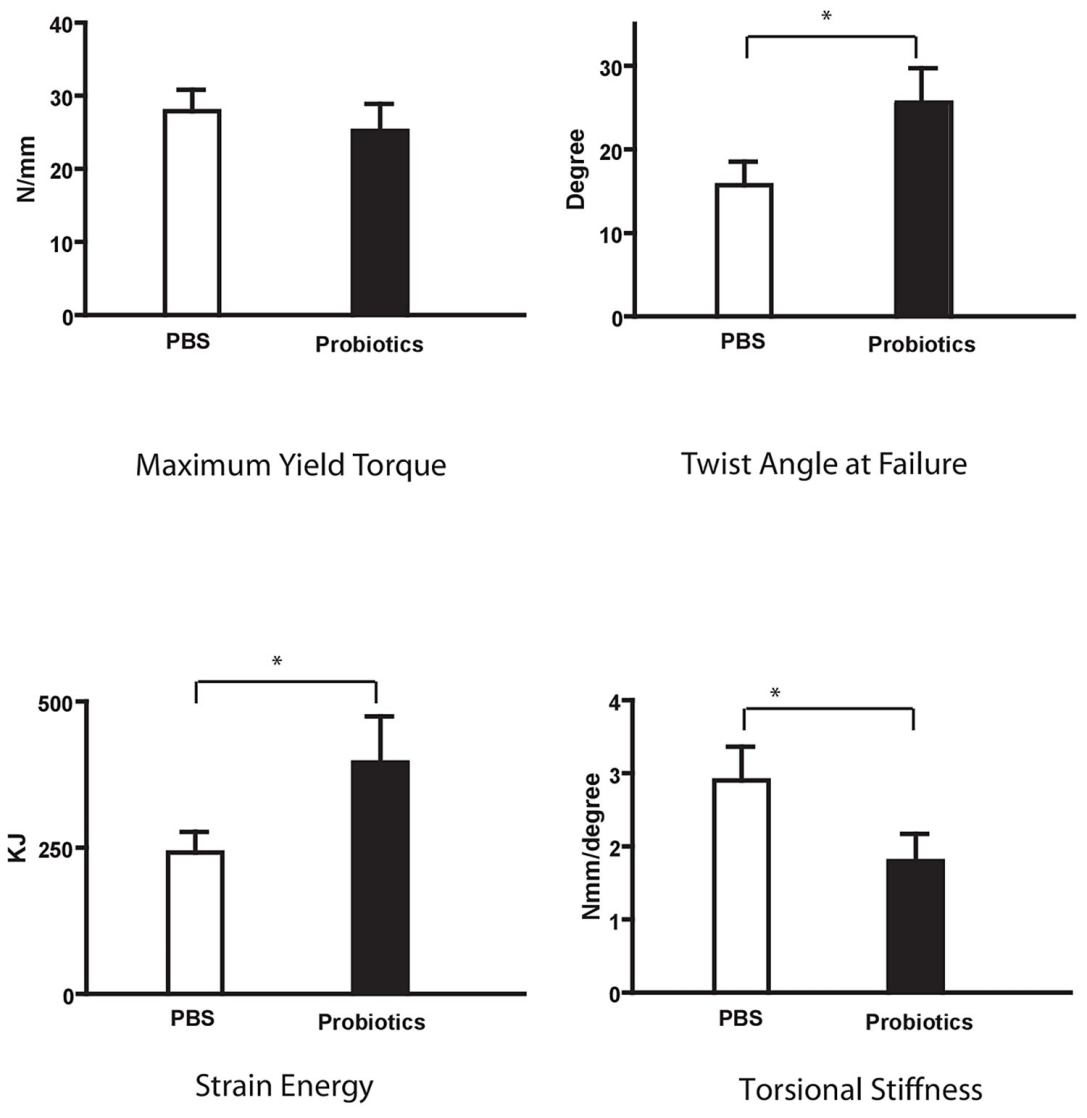
Group 2 biomechanical testing of fractured limb callus at day 28 after fracture–Mechanical assessment of samples showed a higher twist angle and strain energy in the probiotic treatment post-fracture group with a decrease in torsional stiffness *P<0.05.

While there was no difference in maximum yield torque (25.24±3.68 vs 27.90±2.91 N-mm, P = 0.29), the probiotic group demonstrated significantly greater twist angles at failure (25.65±4.10° vs 15.67±2.84°), decreased torsional stiffness (1.79±0.37 vs 2.88±0.46 N-mm/deg) and an increase in strain energy (396.0±78.7 vs 241.9±35.45 KJ) (Fig 7).

## Discussion

Multiple studies have evaluated the possible role of the gut microbiome in bone osteogenesis and regulation as part of development [18] and osteoporosis [19] but few in the setting of fracture. This study shows that daily oral probiotic treatment for 5 weeks pre-fracture in a healthy male mouse fracture model can locally increase cytokine and bone marker gene expression at the fracture site. The fracture callus at day 3 of probiotic treated mice post-fracture expressed significantly higher levels of pro-inflammatory cytokines, IL-6 and IL-17F, compared to untreated controls. We know from previous work [3], that IL-17F is a key pro-inflammatory cytokine enabling the early maturation of osteoblasts to regulate bone regeneration at the cellular level^5^ and is present at the fracture during the early phase of fracture healing. Analysis at day 7 of the fracture callus showed significantly higher gene expression of Runx2 and Col1 in the probiotic treated pre-fracture group 1. This translated to a fracture callus that had more abundant mineralization, a corresponding increase in strength and ability to absorb energy prior to fracture in the probiotic treatment pre-fracture group 1 at 28 days. This data strongly suggests that prior optimization of the gut microbiome with oral probiotics has the potential to improve fracture healing.

Probiotic treatment before an injury occurs is not always possible in the clinical setting. Therefore, assessment of the effectiveness of probiotics given acutely after a fracture occurs was performed. Oral probiotics given post-fracture impacted biomechanical outcomes at day 28 despite no differences noted between groups for transcription of TGF- β, IL-6 and IL-17F levels on day 3 or Col1, Col2 and Runx2 on day 7. It is possible that post-fracture administration may not enable a critical microbiome concentration/equilibrium to be reached early enough to cause a significant change in the inflammatory phase or regulate transcription at days 3–7 to impact the immune mediated stage in bone healing. However, there were differences in the biomechanical properties at day 28 suggesting that critical probiotic levels may have been reached later during the 4-week probiotic treatment post-fracture. The mature callus showed similar volumetric and calcified density profiles and possessed similar maximum yield torque in the treatment and control groups. However, the bone from the probiotic treated post-fracture mice exhibited increased energy strain compared to the control group suggesting higher toughness (reduced stiffness coupled with the increase twist angle at failure despite similar callus volume). This may have arisen due to differences in the callus quality with respect to microarchitecture or material behavior (i.e. the trend towards lower TMD in the treatment group, P = 0.08). Biomarkers assessed in this study such as IL-17F have been established as increasing healing callus ultimate strength [3] but probiotics affect bone metabolism through various mechanisms [7] which may be independent of inflammatory pathways assessed in this study. Nevertheless, callus strain-energy was improved in both pre- and post-fracture treatment settings.

One previous preclinical study assessed fracture healing modulation with probiotic bacterial strains [20] showing that a 2-week treatment of C57BL/6 mice with *bifidobacterium adolescentis*, a bacterium present in normal human gut with known immune-modulation activity, led to tightening of the intestinal barrier, dampened systemic inflammatory response to fracture and accelerated fracture callus cartilage remodeling. Although we did not focus on the mechanistic effects of probiotics on the microbiota in our current mouse fracture model, a recent study demonstrated that probiotics can indeed alter the gut microbiome of C57BL/6 mice and regulate cytokines including IL-10 with increasing bacterial units localizing in the cecum and colon [21]. They also found a protective effect against post-fracture vertebral bone loss and an increase in gut permeability for up to 10 days post-fracture. Similar to our study, no difference in callus size or bone volume were noted, although they reported a transient yet significant reduction in BV/TV at day 14 in the probiotic treated mice. This difference disappeared when assessed 18 days post-fracture and was interpreted as quicker callus remodeling. Mechanical properties of the bone were not assessed. They also noted decreased IL-6 levels in the treatment group, in contrast to our observation of an increase in IL-6. In a subsequent study, the same group found similar results in aged mice, although again only probiotic treatment pre-fracture was investigated [22].

Two clinical studies from one group assessing probiotic use for distal radius fracture, found diet supplementation with *Lactobacillus casei* led to an improvement in functional and pain scores, final radiographic reduction parameters, wrist flexion and grip strength [23,24]. Of note, *Lactobacillus casei* is a strain included in VSL#3 probiotic mixture.

The effect of VSL#3 (and LGG bacterial strain) has been previously assessed in a chemically induced sex steroid–depleted osteoporotic mouse model demonstrating decreased gut permeability and changes in inflammatory mediators with a protective effect against bone loss [25]. These were not seen in germ-free mice or mice treated with E. Coli, a non-probiotic strain, or a mutant LGG. No biomechanical evaluation was performed. Other bacterial strains have been shown to have a beneficial effect on non-fracture bone metabolism such as *Lactobacillus reuteri* [26,27], *paracasei* [28], and *Bifidobacterium longum* [29]. In one study, an inflammatory insult was required for the effect of the probiotics to be observed [30]. Divergences between the result of this and other studies can likely be explained by varying experimental models (non-fracture setting, the use of a specific bacterial strain vs a mixture, host mice genotype background and different baseline gut microbiome [30]).

This study has the following limitations. A healthy young mouse model (11–15 weeks of age) with robust healing potential was used. Previous studies have shown that C57BL/6 mice only start exhibiting an osteoporotic phenotype at ~1 year. It may be beneficial to evaluate the impact of probiotics using an impaired fracture healing model such as an older, ovariectomized or osteoporotic model [31]. Biochemical analysis was limited to transcription which does not necessarily correlate 1:1 with protein synthesis or molecule activity although some general correlation would be expected. This study also assessed solely diaphyseal bone healing for the purposes of facilitating mechanical testing; the impact of probiotics may be different for metaphyseal fracture healing [32].

This study was not designed to assess ideal probiotic titration quantity or timing. Primary callus formation (days 7–9 in mice) and mineralization (days 7–21) are distinct temporal steps in fracture healing [33]. Modulating the gut microbiome within a specific timeframe may be critical for optimal results, as shown with other cytokines affecting bone healing [15]. Also, this study evaluated secondary bone healing through endochondral ossification. Evaluation in the setting of rigid fixation with absolute stability may help further elucidate the contribution of the gut microbiome to different aspects of bone healing.

VSL#3 is a mixture of 8 different bacterial strains, and its effectiveness has been evaluated in multiple clinical studies [34], primarily focused on gastrointestinal-related diseases. It is unclear if a subset of the VSL#3 bacteria, a specific strain or simply a change in the microbiome played a critical role in the effects seen on fracture healing. Elucidating specific active bacteria could lead to a more targeted mixture with stronger beneficial effects. Hence, future studies will include the identification specific bacterial organisms of probiotic treatment through fecal specimen 16s rRNA or metagenomic sequencing analysis. However, VSL#3 is a commercially available product making it amenable for rapid low cost, low risk transition to use in a clinical setting. Probiotics are components of multiple dietary foods such as yogurt that are widely ingested by many and may be readily accepted by patients and practitioners.

This study suggests that oral probiotic administration may alter the gut flora microenvironment to stimulate inflammatory cells at the fracture site to up-regulate the expression of inflammatory cytokines and bone markers essential to bone healing. Treatment of mice with probiotic VSL#3 pre- and post- fracture led to positive changes in the healed bone biomechanical properties. This is a novel finding that increases the attractiveness of probiotic use in the clinical setting as an acute treatment to possibly improve fracture healing or prevent re-fracture, particularly in the setting of osteoporosis or pathological bone where future fracture risk is high. This requires further investigation as new adjunct therapies are imminently needed to alleviate the burden of managing fractures in patients and on the healthcare system. Further work may lead to the use of probiotics as a cost-effective and low-risk adjunctive therapy to improve bone fracture healing.

## Supporting information

S1 File (XLS)

S2 File (XLS)

S3 File (XLS)

S4 File (XLSX)

S5 File (XLSX)

S6 File (XLSX)

S7 File (XLSX)
